# Parkinsonism in complex neurogenetic disorders: lessons from hereditary dementias, adult-onset ataxias and spastic paraplegias

**DOI:** 10.1007/s10072-023-07044-9

**Published:** 2023-08-30

**Authors:** Simone Aloisio, Sara Satolli, Gabriele Bellini, Piervito Lopriore

**Affiliations:** 1 Department of Advanced Medical and Surgical Sciences (DAMSS), University of Campania “Luigi Vanvitelli”, Naples, Italy; 2 Molecular Medicine for Neurodegenerative and Neuromuscular Diseases Unit, IRCCS Fondazione Stella Maris, Pisa, Italy; 3 Department of Clinical and Experimental Medicine, Neurological Institute, University of Pisa, Pisa, Italy

**Keywords:** Neurogenetics, Parkinsonism, Familial Alzheimer’s disease, Frontotemporal dementia, Hereditary ataxia, Hereditary spastic paraplegia

## Abstract

Parkinsonism is a syndrome characterized by bradykinesia in combination with either rest tremor, rigidity, or both. These features are the cardinal manifestations of Parkinson’s disease, the most common cause of parkinsonism, and atypical parkinsonian disorders. However, parkinsonism can be a manifestation of complex neurological and neurodegenerative genetically determined disorders, which have a vast and heterogeneous motor and non-motor phenotypic features. Hereditary dementias, adult-onset ataxias and spastic paraplegias represent only few of this vast group of neurogenetic diseases. This review will provide an overview of parkinsonism’s clinical features within adult-onset neurogenetic diseases which a neurologist could face with. Understanding parkinsonism and its characteristics in the context of the aforementioned neurological conditions may provide insights into pathophysiological mechanisms and have important clinical implications, including diagnostic and therapeutic aspects.

## Introduction

Parkinsonism is one of the most common hypokinetic movement disorders worldwide. According to MDS clinical diagnostic criteria of 2015, parkinsonism is defined by the presence of bradykinesia in combination with either rest tremor, rigidity, or both [1]. Although its presence is the core feature of idiopathic and monogenic Parkinson’s disease (PD) or atypical parkinsonian diseases including progressive supranuclear palsy (PSP), corticobasal syndrome (CBS), multiple system atrophy (MSA) and dementia with Lewy bodies (DLB), parkinsonism is frequently reported as part of a more complex neurogenetic disorders’ phenotypic spectrum. For a clinical point of view, it should be highlighted that the current definition of bradykinesia does not necessarily apply to all cases of parkinsonism. The term bradykinesia, which means slowness of movement, is used interchangeably to indicate hypokinesia (low amplitude movement) or akinesia (absence of movement). Recently, Bologna and colleagues proposed a redefinition of bradykinesia (bradykinesia-complex), suggesting that parkinsonism can be diagnosed in the presence of a combination of motor alterations, that is, bradykinesia with sequence effect plus any additional features [2].

Neurogenetic disorders are defined as “clinical diseases caused by a defect in one or more genes, which affects the differentiation and function of the neuroectoderm and its derivates”. They represent a wide spectrum of diseases, most of which are rare (prevalence < 5 cases per 100.000 persons in European Union) [3]. Parkinsonism may represent a motor phenotype in the complexity of these disorders: hereditary dementias, ataxias, spastic paraplegias, neurodegeneration with brain iron accumulation, leukodystrophies, congenital metabolic disorders, neuromuscular diseases, mitochondrial disorders, epileptic encephalopathies represent only few of this vast group.

**Fig. 1:**
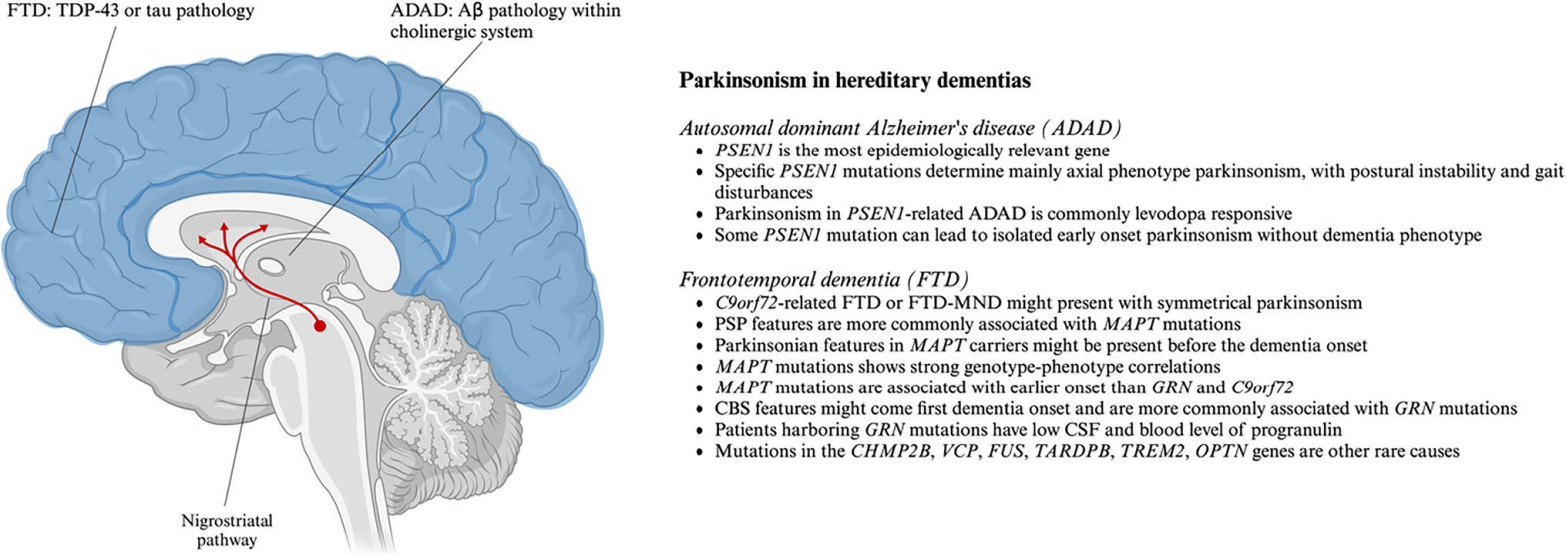
Parkinsonism in hereditary dementias - key points

**Fig. 2:**
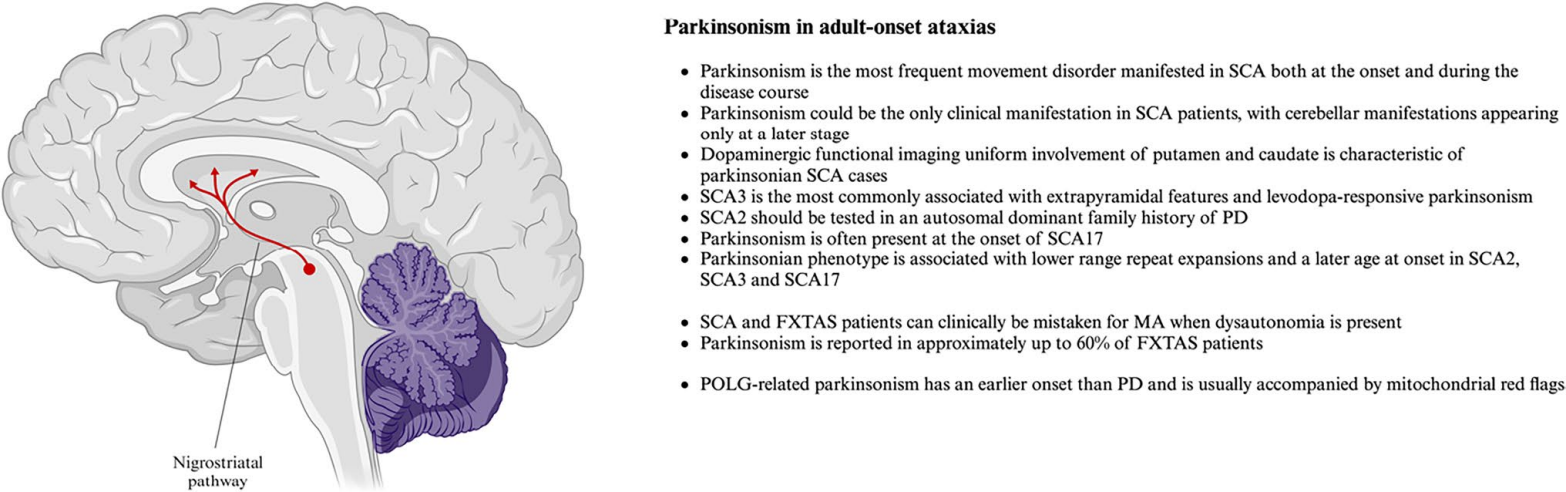
Parkinsonism in adult-onset ataxias - key points

**Fig. 3:**
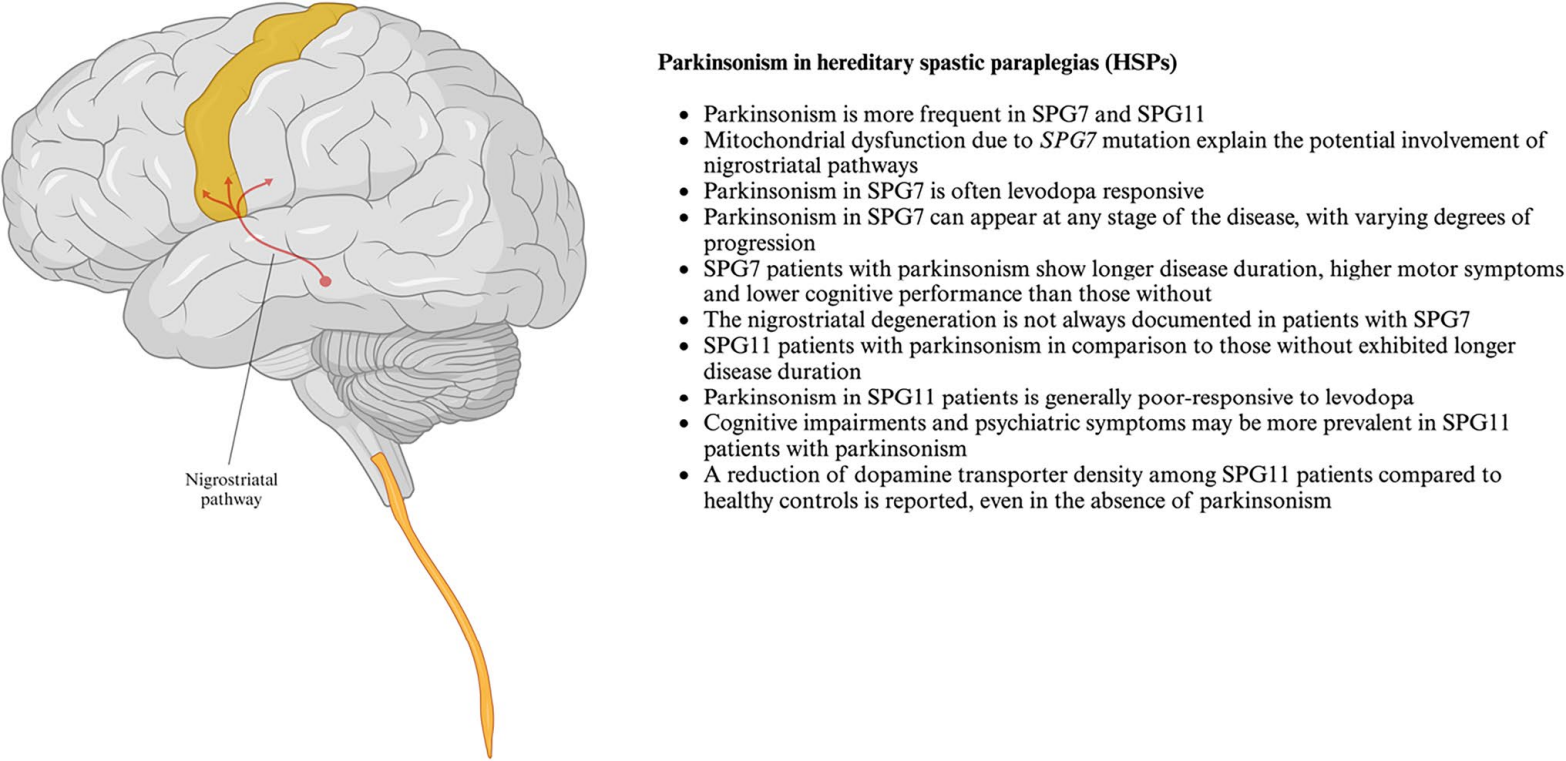
Parkinsonism in hereditary spastic paraplegias - key points

This narrative review aims to provide an overview of parkinsonism’s clinical features within the phenotypic spectrum of some of the most common adult-onset neurogenetic disorders, in particular hereditary dementias, adult-onset ataxias and spastic paraplegias. For each disease group discussed a summary image with key points is provided (Fig. 1, 2, and 3). Finally, Table 1 summarized the main clinical and imaging features of parkinsonism in the complex neurogenetic disorders described.

## Hereditary dementias

### Familial Alzheimer's disease

Parkinsonism frequently accompanies Alzheimer disease (AD), especially when it starts at an early age [4]. In early onset AD, autosomal dominantly inherited AD (ADAD) accounts for 5–10% of cases and commonly exhibits various motor symptoms such as parkinsonism, dystonia, cerebellar ataxia and spastic paraparesis [5, 6]. ADAD is caused by mutations in either presenilin 1 (*PSEN1*), presenilin 2 (*PSEN2*), or amyloid precursor protein (*APP*) gene. A large body of evidence supports a link between AD and parkinsonism. Deposits of α-synuclein are frequently observed in AD and Lewy bodies were described in the substantia nigra of familial AD [7]. Among known amyloidogenic genes potentially leading to parkinsonism, *PSEN1* is the most epidemiologically relevant. To date, parkinsonian features in *APP*- or *PSEN2*-related ADAD have not been identified. Parkinsonism in *PSEN1* carriers shows asymmetrically decreased dopamine transporter (DaT) PET uptake in the striatum and has a global favorable response to levodopa treatment [8]. Heterozygous mutations in *PSEN1* (p.Phe105Leu, p.Met146Val, p.Glu280Ala, p.Leu286Val, p.Leu392Val) are responsible for familial AD, with parkinsonism mainly appearing in advanced stages. While frequently observed in late phases, parkinsonism at onset with mainly axial phenotype and presenile dementia has been associated with specific *PSEN1* mutations (p.Gly217Asp and p.Val272Ala) [9, 10]. In these forms with predominant axial signs such as on-period freezing, increased gait variability, and postural instability, dopaminergic therapy is not effective. Interestingly, one of the underlying mechanisms of gait disorders in parkinsonism is a cholinergic deficit resulting from amyloid-β (Aβ) pathology within cholinergic pathways (pedunculopontine nucleus, thalamus, nucleus basalis of Meynert and the forebrain cortical projections) [11, 12]. *PSEN1* mutations can also determine early-onset “pure” parkinsonism, without dementia phenotype. Gatto and colleagues reported a novel *PSEN1* mutation (p.Arg41Ser) in an Argentinian patient presenting with a motor phenotype characterized by good levodopa response, presence of levodopa induced dyskinesia, normal cognitive function in the absence of standard AD biomarkers (MRI moderate frontal cortical atrophy, negative research for cortical amyloid deposition and 18F-FDG-PET mild hypometabolism in left lateral temporal lobe) [13].

In the last decade the number of *PSEN1* mutations discovered has increased; complex neurological phenotypes such as seizures, myoclonus, spastic paraparesis and ataxia, in variable associations with cognitive impairment and parkinsonism, have been reported. For example, patients carrying the p.Ser170Phe mutation may display a different phenotype including myoclonus, seizures and cerebellar ataxia [7, 14]. In 2017, Carecchio and colleagues reported on a case of a de novo yet unknown *PSEN1* mutation (p.Ser170Phe) presenting with a predominant motor phenotype, an early-onset dystonia-parkinsonism, later complicated by dementia and myoclonus, with brain MRI mimicking basal ganglia iron accumulation [15].

Although overt parkinsonism is associated with *PSEN1* mutations, the underlying pathomechanism is unclear. Parkinsonism might be related to presynaptic or postsynaptic dopaminergic dysfunction. Presynaptic dopaminergic dysfunction in ADAD may be explained by α-synuclein or Aβ pathology in substantia nigra. Autopsy studies have shown that AD patients with parkinsonism have more neurofibrillary tangles in the substantia nigra than those without parkinsonism [16]. Vice versa, other studies have suggested that postsynaptic dopaminergic dysfunction is responsible for parkinsonism in AD, with early deposition of amyloid in the striatum of ADAD patients [17]. Moreover, it has also become apparent that some *PSEN1* mutations are associated with neuropathological lesions not typically observed in AD. One of these lesions is the so-called ‘cotton wool’ plaque (CWP) that has been found associated with several *PSEN1* mutations. Severe involvement of the caudate nucleus and putamen with CWPs may play a critical role in the severity of *PSEN1* related parkinsonian syndrome [9]. These observations are clinically relevant because they suggest that dopaminergic replacement in early-onset or in familial AD does not necessarily improve motor symptoms in this condition. Clinicians should carefully consider levodopa when parkinsonism is associated with presynaptic dopaminergic deficit.

### Frontotemporal dementia

Frontotemporal dementia (FTD) is one of the main causes of dementia among the population under 65 years or age. The term FTD encompasses various clinical-pathological forms of hereditary neurodegeneration, some of which are rare if taken at single level. FTD manifests mainly with alterations in behavior, language, or executive functions resulting from frontotemporal lobar degeneration (FTLD), a set of neuropathologic changes primarily occurring at the frontal and temporal lobes of the brain. From a clinical point of view, FTD encompasses two main syndromes: behavioural variant FTD (bvFTD) and primary progressive aphasia (PPA) [18]. Overall, about 40% of FTD patients have a positive family history of dementia, psychiatric diseases, or motor symptoms (motor neuron disease or parkinsonism) with 10% of them showing autosomal dominant inheritance pattern. To date, mutations in microtubule-associated protein tau (MAPT), progranulin (GRN) and chromosome 9 open reading frame 72 (C9orf72) account for the majority of genetically determined FTD (about 30%) [19].

Patients with FTD can present either parkinsonism or atypical parkinsonism, the latter manifesting as a CBS, or PSP spectrum [20]. Indeed, CBS and PSP, which can show in their clinical course signs of frontal lobe or language dysfunction, are classified as FTD-related disorders reflecting their underlying tau pathology [18]. Overall, parkinsonism is the most common movement disorder in patients with bvFTD (60–80% of patients), rarely occurring in the other clinical forms. It usually presents, regardless of the genetic aetiology, as symmetrical rigid-akinetic syndrome, meanwhile typical resting tremor is rare. Usually, levodopa response is poor or transitory (especially in the initial stage of the disease). The development of parkinsonism in FTD is the result of the functional and structural degeneration of various brain structures implicated in motor control. Particularly, presynaptic and postsynaptic dopaminergic failure have been demonstrated in FTD patients manifesting parkinsonism, as well as reduced glucose metabolism in subcortical brain areas. Moreover, basal ganglia atrophy has been attributed to the loss of afferent inputs from the affected cortical areas [20–22]. Interestingly, abnormal primary motor cortex excitability, long-term potentiation and long-term depression-like plasticity were found in CBS and FTD (both bv-FTD and PPA) patients manifesting parkinsonism. These neurophysiological findings likely reflect the neurodegenerative processes involving the cortico-basal ganglia-thalamo-cortical motor loops [23, 24].

Parkinsonism was first observed in patients with FTD linked to chormosome 17 in the '90 s (FTDP-17). In subsequent years it turned out that these patients carried mutations in MAPT or in GRN [25]. So far, C9orf72 hexanucleotide intronic expansion is known to be a major causative cause of FTD and FTD and motor neuron disease (FTD-MND). These three main genetic causes will be overviewed. However, mutations in other genes (CHMP2B, VCP, FUS, TARDPB, TREM2, OPTN) have been identified in a minority of FTD patients with parkinsonian features [19, 25].

#### MAPT

*MAPT*, located in chromosome 17q21.31, encodes for microtubule-associated protein tau. To date, more than 80 mutations in *MAPT* gene have been associated with different tauopathies [26]. Most of the mutations linked to FTD and parkinsonism are located in the exon 10 [21]. These patients usually present with bvFTD and parkinsonism with an early age of onset (early 40 s). Parkinsonism can precede the behavioral/cognitive symptoms of FTD (usually 10 years earlier) or occur in the later course of the disease [20]. There is increasing evidence of a strong genotype–phenotype correlation. Some variants, indeed, are associated with early-onset, poor levodopa responsive, aggressive parkinsonism (i.e. p.Asn279Lys, less commonly c.915 + 16C > T), other with a milder or late onset form (i.e. p.Ser305Asn) [20]. Moreover, some variants (i.e. p.Leu284Arg, p.Asn296del, p.Gly303Val, p.Lys317Met, c.915 T > C and c.853A > C) have been correlated with bvFTD associated with PSP features, such as axial rigidity and supranuclear palsy, or, more rarely, with CBS-like presentation or motor neuron disease features [27, 28]. However, clinical variability has been observed in individuals harboring the same mutations, in different families or even within the same family [28].

#### GRN

*GRN*, located near *MAPT* locus, encodes for the glycoprotein progranulin. Thus far, more than 70 different mutations have been described, all of them causing FTD via haploinsufficiency and manifesting as TDP-43 pathology [29, 30]. Early onset (early 50 s) bvFTD and nonfluent variant PPA are the commonest phenotypes. Compared to *MAPT*, overall clinical variability seems to be wider in *PRGN*-related FTD with weaker genotype–phenotype correlations [19, 27, 31]. Parkinsonism, usually rigid-akinetic, is common as additional FTD-following manifestation (25–40%), but rare as presenting features. Tremor is less common (< 5%), whereas hallucinations occur more frequently than in those patients carrying other FTD-related gene mutations [20, 32]. Higher incidence of parieto-occipital asymmetric interhemispheric atrophy leading to CBS phenotype (asymmetric apraxia and dystonia, visuospatial dysfunction) has been observed (even presenting early in the disease course), especially in those harboring a 4 bp deletion in exon 7 (p.Leu271LeufsX10) [33]. PSP spectrum phenotype is a rarer manifestation [20, 21, 32]. 123I­FP­CIT SPECT revealed an asymmetric reduction in tracer uptake in a patient harboring GRN mutation [34].

#### C9orf72

Hexanucleotide GGGGCC repeat expansion in the first intron of *C9orf72* gene, located in chromosome 9q21-q22, is the commonest genetic cause of FTD, ALS and FTD-MND in Europe and North America [18]. Up to thousands hexanucleotide repeats are associated with disease. C9orf72 protein play pivotal roles in intracellular endosomal trafficking and autophagy. *C9orf72* expansion carriers present TDP-43 positive inclusion on brain autopsy [35, 36]. Parkinsonism is reported in about 30–35% of patients with *C9orf72* expansion, usually associated with early onset (early 50 s) bvFTD, FTD-MND or, more rarely, pure ALS [20, 21, 25]. Beyond the commonest symmetric akinetic-rigid syndrome, parkinsonism may present as asymmetric syndrome with or without postural or resting tremor or gait ataxia and autonomic disturbance, mimicking MSA [37]. Cases presenting with CBS or PSP phenotypes have rarely been described [20, 21, 27]. Intermediate (20–30) *C9orf72* repeat lengths do not influence FTD (or PD, atypical parkinsonism, and ALS) disease risk [38]. 123I­FP­CIT SPECT and post-mortem analysis revealed symmetric pre-synaptic dopaminergic disturbance in *C9orf72* expansion carriers [25].

## Hereditary adult-onset ataxias

Hereditary ataxias are a clinically and genetically heterogeneous group of disorders characterized by slowly progressive incoordination of gait often associated with poor coordination of hands, speech, and eye movements [39]. The number of genetic loci associated with inherited ataxias is rapidly growing [40]. Inheritance can be autosomal dominant, autosomal recessive, X-linked or maternal if part of a mitochondrial genetic syndrome [39]. Peculiar anamnestic, clinic and radiologic features should guide the correct diagnosis. Among hereditary ataxias, parkinsonism is frequently part of the clinical spectrum or even the only manifestation.

### Spinocerebellar ataxias

Spinocerebellar ataxias (SCAs) are an inherited autosomal dominant, neurodegenerative, and heterogeneous group of disease that mainly affects the cerebellum. SCAs are in most cases caused by pathogenic expansions of short tandem CAG repeats (so termed SCA-polyQ forms). CAG repeats tend to progressively expand in consecutive generations causing anticipation of the age at onset. The phenotypic spectrum of SCAs is broad and ranges from pure cerebellar ataxia to more complex clinical pictures [41]. Parkinsonism is the most frequent movement disorder manifested in SCAs both at the onset and during the disease course. The akinetic-rigid phenotype tends to be more frequent than the tremor-dominant one [42]. Symmetrical or asymmetric involvement, late onset, slow progression, and good response to dopaminergic treatment in half of the cases resemble PD. Motor fluctuations and dyskinesias have also been described in a few of patients [41]. Interestingly, the motor phenotype may remain purely extrapyramidal, with cerebellar manifestations appearing only at a later stage [42]. On MRI, only one third of patients with isolated parkinsonism exhibited cerebellar or brainstem atrophy [41]. Pre- and postsynaptic dopaminergic functional imaging can show predominant and asymmetric putaminal involvement, as those observed in PD, or uniform affectation of putamen and caudate more characteristic of parkinsonian SCAs cases [41]. Screening for SCAs mutations in patients with parkinsonism without a cerebellar syndrome is not recommended, except for familial cases [42]. Prominent parkinsonism can be a typical manifestation in SCA2, SCA3, and SCA17, even though it has been described in almost all SCAs subtypes [42]. In all three, parkinsonian phenotype is associated with lower range repeat expansions and a later age at onset [42].

SCA2, caused by a CAG expansion in *ATXN2* gene, generally present as slowly progressive cerebellar ataxia variably associated with peripheral neuropathy, dystonia, myoclonus, autonomic dysfunction, and slowed horizontal saccades. In a subset of patients, levodopa-responsive rest tremor, bradykinesia, and rigidity, are the only manifestations, with mild ataxic features manifesting in later stages. Therefore, SCA2 should be considered in the differential diagnosis of PD patients with an autosomal dominant family history [43]. Contrariwise, when family history is negative, the combined presence of cerebellar ataxia, parkinsonism, and autonomic dysfunction, can clinically be mistaken for MSA.

SCA3 (CAG-*ATXN3*), the most common SCA worldwide, is probably also the most commonly associated with extrapyramidal features and levodopa-responsive parkinsonism [42]. In SCA3 patients, cerebellar syndrome is variably accompanied by pyramidal signs, peripheral neuropathy, ophthalmoplegia and dystonia.

The diagnosis of SCA17 is established by identification of an abnormal CAG/CAA repeats in *TBP* gene. SCA17 may express pleiotropic neurological manifestations, rages from cerebellar ataxia, cognitive impairment, psychiatric symptoms, and a wide spectrum of different movement disorders. Chorea, dystonia, and akinetic-rigid parkinsonism are the most representative. Parkinsonism is often present at the onset [41]. Considering that parkinsonism is frequent in SCAs, the extrapyramidal signs should not be overlooked in these patients, and a therapeutic attempt with levodopa should be tried since a good response has been described in some patients.

### FXTAS

Fragile X tremor-ataxia syndrome (FXTAS) is a late-onset neurodegenerative disorder, associated with the fragile X mental retardation 1 (*FMR1*) gene premutation (55–200 CGG repeat expansion) [44]. The penetrance of FXTAS in male carriers over 50 years old is about 40%, whereas female carriers tend to have a milder phenotype with ovarian dysfunction and psychiatric symptoms. The typical phenotype consists of the combination of intention tremor and ataxia [44]. Parkinsonism is reported in approximately up to 60% of patients, with rigidity and bradykinesia being the most prevalent of the parkinsonian symptoms [44, 45]. Rest tremor, postural instability, autonomic dysfunction, and cognitive decline have also been described [45]. A positive dopaminergic treatment response is sometimes present [45, 46]. Typical MRI features are increased signal intensity in the middle cerebellar peduncles (MCPs) on T2-weighted images or FLAIR sequences, also known as MCP sign, and white matter hyperintensities in the splenium of the corpus callosum [47]. Considering the phenotypic spectrum of these patients, the coexistence of parkinsonian syndrome and dysautonomia, FXTAS should be considered in the differential diagnosis of MSA [48]. The presence of early cognitive dysfunction, a family history of mental retardation or premature ovarian failure, the presence of MCP sign provide important clues. Conversely, DaTscan is not useful. Abnormal nuclear imaging has been reported in approximately 47% of patients with FXTAS, specifically those with MSA–like features [49].

### POLG-related disorders

Mitochondrial dysfunction has been implicated in the pathogenesis of idiopathic and monogenic PD; thus, it is not surprising that mutations of genes encoding mitochondrial proteins like the nuclear-encoded mtDNA polymerase gamma (*POLG*) cause a parkinsonian syndrome among their clinical manifestations [50]. POLG mutations cause a wide spectrum of overlapping phenotypes including ataxia, seizures, myopathy, progressive external ophthalmoplegia, as well as several movement disorders. Onset of *POLG*-related disorders ranges from infancy to late adulthood [51]. Ataxia in *POLG*-related disease can be sensory or cerebellar and it seems to be a frequent cause of ataxia in Central Europe once repeat expansion diseases had been excluded [51]. Parkinsonism is the most frequently observed extrapyramidal movement disorder in patients with *POLG* mutations and has been associated with both dominant and recessive mutations [48]. *POLG*-related parkinsonism has an earlier onset than PD typically ~ 40 years but even earlier [51, 52]. Parkinsonism is usually accompanied by typical mitochondrial disease signs and symptoms such as short stature, neurosensory hearing loss, ptosis, ophthalmoplegia, axonal neuropathy, diabetes mellitus, hypertrophic cardiomyopathy, renotubular acidosis, migraine-like headache [53]. In all cases, patients had a positive DaTscan with reduced striatal uptake bilaterally and a sustained response to dopaminergic treatment [51]. Levodopa-induced dyskinesias and motor fluctuations may also occur [49]. Intriguingly, other nuclear-encoded mitochondrial genes regulating mtDNA replication and maintenance, such as *OPA1* and *TWNK* have been correlated with parkinsonism. Parkinsonism may be an additional neurological feature in the autosomal dominant optic atrophy “plus” phenotype or, in some cases, be the cardinal manifestation without evidence of clinical visual disturbances [54, 55]. Regarding *TWNK*, some reports of parkinsonism in *TWNK*-related autosomal dominant progressive external ophthalmoplegia have been described in the literature so far [56].

## Hereditary spastic paraplegias

Hereditary spastic paraplegias (HSPs) encompass a heterogeneous group of neurodegenerative disorders with a prevalence ranging from 2 to 7.4 per 100,000 individuals in most populations. These conditions can be inherited in different patterns, including autosomal dominant, autosomal recessive, or X-linked, and their onset can occur anywhere from early childhood to 70 years of age [57–59]. Alongside the characteristic spastic paraplegia, an increasing body of evidence suggests that individuals with HSP may also present with parkinsonism-like symptoms. Parkinsonism, resembling features seen in PD, adds a layer of complexity to the clinical presentation of HSP. This paragraph will focus on exploring the specific features of parkinsonism observed in Spastic paraplegia type 7 (SPG7) and Spastic paraplegia type 11 (SPG11), two subtypes of HSP. Despite Spastic paraplegia type 4 being the most frequent form of HSP, recent studies have shown the emergence of parkinsonism as one of the symptoms to investigate, especially in SPG7 and SPG11, two HSPs with autosomal recessive transmission [60, 61]. In addition, there are also reports of parkinsonism in other HSP variants, such as Spastic paraplegia type 30 (involving the *KIF1A* gene) and Spastic paraplegia type 35 (involving the *FA2H* gene), although these are less common [62, 63].

### SPG7

The *SPG7* gene, spanning about 52 kb and consisting of 17 exons, is located on chromosome 16q24.4 and encodes the protein paraplegin, a mitochondrial metalloprotease present in the inner mitochondrial membrane [64]. Paraplegin plays a crucial role in various mitochondrial processes, including the surveillance of mitochondrial protein quality and protein dislocation [65]. SPG7 is an autosomal recessive HSP characterized primarily by ataxia, which can be found in up to 57% of patients. The condition is marked by progressive bilateral lower limb weakness and spasticity. Additionally, one-third of SPG7 cases exhibit cerebellar abnormalities on MRI [66]. Other common symptoms may include spastic dysarthria, dysphagia, ophthalmic findings like nystagmus, strabismus, ptosis, pale optic discs, and urinary sphincter disorders [66].

In animal models, *SPG7* is intensely expressed in Purkinje cells and moderately in the striatum, suggesting possible involvement of nigrostriatal pathways in patients with *SPG7* mutations [67]. Furthermore, the clinical spectrum of SPG7 continues to broaden, and recent years have seen a connection between *SPG7* mutations and parkinsonism [68]. Misdiagnosis with extrapyramidal syndromes, especially MSA type, has been reported [69, 70]. Moreover, large genetic databases have revealed a significantly higher frequency of p.Ala510Val variant carriers among PD patients, indicating SPG7 as a potential candidate gene for early-onset PD [71]. In 2018, Magri and colleagues described a unique phenotype characterized by early-onset optic atrophy, spastic ataxia, and levodopa-responsive parkinsonism associated with a de-novo heterozygous mutation of *AFG3L2* (p.Arg468Cys) and a maternally inherited heterozygous intragenic deletion of *SPG7*. This complex phenotype could not be solely attributed to both genes individually [72]. In a study evaluating parkinsonism in 33 patients with pathogenic *SPG7* mutations, De La Casa-Fages and colleagues found 7 patients with parkinsonian signs, with bradykinesia being the most frequently observed. One patient had parkinsonism complicated by Pisa syndrome, and all three patients treated with levodopa responded positively to the treatment. The onset of parkinsonism varied, appearing either at the beginning or during the disease, without clear progression over time. Among three patients who underwent 123I­FP­CIT SPECT, one showed bilateral presynaptic denervation, another had mild unilateral denervation, and the third patient's scan was normal [54]. Additional reports described the same clinical features of parkinsonism in patients with *SPG7* mutations [66, 73].

### SPG11

SPG11 is a rare genetic neurodegenerative condition characterized by progressive spasticity in the lower limbs. However, recent evidence suggests that some patients with SPG11 may also develop parkinsonism [56]. In these cases, parkinsonism manifests as an additional motor phenotype alongside spastic paraplegia. Patients with SPG11-related parkinsonism typically experience a more severe disease progression and an earlier onset compared to those without parkinsonism. Additionally, cognitive impairments and psychiatric symptoms, including depression and anxiety, may be more prevalent in individuals with SPG11 and parkinsonism [60, 74]. The exact mechanisms linking SPG11 and parkinsonism are not yet fully understood. SPG11 arises from mutations in the *SPG11* gene, which encodes spatacsin, a protein involved in the lysosomal trafficking pathway. Dysfunction of this pathway in *SPG11* results in impaired protein degradation and the accumulation of toxic aggregates, leading to neurodegeneration [75].

Among the various reported cohorts of SPG11, one of the most extensive studies highlighted the presence of parkinsonian features in 5 out of 30 cases, indicating an approximate prevalence of 16.7% Interestingly, within this subgroup, 60% of individuals demonstrated a positive response to levodopa treatment [74]. However, it is worth noting that other larger series of SPG11 patients did not report the occurrence of parkinsonism [76].

Neuroimaging studies have provided valuable insights into SPG11-related parkinsonism. Reduced binding of DaT has been observed in the striatum, suggesting impaired dopaminergic function [77].

Faber and colleagues examined in vivo the dopaminergic system in a cohort of 22 SPG11 patients in patients with and without parkinsonism through the 99mTc-TRODAT-1 SPECT. They identified 6 patients with parkinsonism (27.3%) mainly rigid-akinetic symmetric. Patients with parkinsonism in comparison to those without parkinsonism exhibited longer disease duration, higher scores on the Spastic Paraplegia Rating Scale (SPRS), and lower cognitive performance on the Montreal Cognitive Assessment (MoCA). Lower DAT densities were observed in the left caudate in parkinsonian patients, with a tendency towards lower DAT densities in the striatum. However, they noted a universal reduction of DAT density among SPG11 patients compared to healthy controls, even in the absence of parkinsonism [60]. In their cohort only a proband had a meaningful improvement in bradykinesia with levodopa treatment, but he had to withdraw because of worsening of previous psychotic symptoms [60]. These neuroimaging findings contribute to the understanding of the disease's pathophysiology, aid in accurate diagnosis, and potentially serve as biomarkers for monitoring disease progression and treatment response.

## Conclusions

Parkinsonism is one of the most prevalent movement disorders among complex neurogenetic diseases. The emergence of parkinsonism in complex neurogenetic patients adds complexity to the clinical presentation of this rare inherited neurodegenerative disorder. Parkinsonism could represent part of the disease core-phenotype or being a secondary manifestation of the disease spectrum, preceding or accompanying the core features (Table 1).

**Table 1:** Main clinical and imaging features of patients with parkinsonism in complex neurogenetic disorders

	Familial Alzheimer’s disease	Frontotemporal dementia			Hereditary adult-onset ataxias			Hereditary spastic paraplegias	
Genotype/phenotype	*PSEN1*	*MAPT*	*GRN*	*C9orfF72*	SCA	FXTAS	*POLG*	SPG7	SPG11
Parkinsonism at onset	±	±	-	±	+	±	+ +	+	-
Rigid-akinetic parkinsonism	+ +	+ + +	+ + +	+ +	+ +	+ +	+	+	+ +
Levodopa response	+ +	+	+	+	+ +	+	+ + +	+ +	±
Nigrostriatal degeneration	+ + Asymmetric	+ Symmetric	+ Asymmetric	+ Symmetric	+ + Asymmetric	+ +	+ + + Symmetric	+ Symmetric	+ + Symmetric

- uncommon, + reported, +  + common, +  +  + frequent, ± uncertain

After the onset of a non-parkinsonian phenotype with a specific molecular diagnosis or a high suspicion of neurogenetic disease, special attention should be paid to looking for the emergence or subclinical presence of any signs indicative of possible parkinsonism (bradykinesia, usually symmetric rigidity, rarely tremor). In the case of FTD, elements of CBS (i.e. asymmetric bradykinesia-rigidity-dystonia) or PSP (slowness of saccades, limitations in upward gaze, rigidity, unsteadiness in walking) should be checked. Parkinsonism should be carefully investigated, especially why in some disorders or specific genotype (e.g. SCAs, POLG-related ataxias, some *PSEN1* mutations, SPG7) a good levodopa response has been reported.

Intriguingly, for some specific mutations in genes related to other phenotypes (e.g. hereditary dementias, SCAs, *POLG*-related ataxias, SPG7) parkinsonism may even represent the core clinical manifestation at the onset, making molecular diagnosis difficult. In these cases, more attention should be paid to the presence of subclinical or later emergence of unexpected signs/symptoms (such as cerebellar ataxia, pyramidal signs, progressive external ophthalmoplegia, mitochondrial red flags). These elements, added to a slow progression of extrapyramidal symptoms, could offer a cue to question the diagnosis of PD.

Although precise mechanisms connecting parkinsonism to these complex neurogenetic disorders are not fully elucidated, patients need comprehensive and tailorized management approaches. Further research is warranted to deepen our understanding of complex neurogenetic disorders-related parkinsonism and develop targeted interventions to improve the quality of life for affected individuals.

## Data Availability

No new data were created or analysed in this study. Data sharing is not applicable to this article. All the literature used for this review is listed in the bibliography.

## Abbreviations

*PD*: Parkinson’s disease
*PSP*: Progressive supranuclear palsy
*CBS*: Corticobasal syndrome
*MSA*: Multiple system atrophy
*DLB*: Dementia with Lewy bodies
*AD*: Alzheimer’s disease
*ADAD*: Autosomal dominantly inherited
*PSEN1*: Presenilin 1
*PSEN2*: Presenilin 2
*APP*: Amyloid precursor protein
*Aβ*: Amyloid-β
*MRI*: Magnetic resonance imaging
*DAT*: Dopamine transporter
*18F-FDG-PET*: 2-Deoxy-2-[fluorine-18]fluoro- D-glucose positron emission tomography; cotton wool’ plaque
*FTD*: Frontotemporal dementia
*FTLD*: Frontotemporal lobar degeneration
*bvFTD*: Behavioural variant FTD
*PPA*: Primary progressive aphasia
*MAPT*: Microtubule-associated protein tau
*GRN*: Progranulin
*C9orf72*: Chromosome 9 open reading frame 72
*FTD-MND*: Frontotemporal dementia and motor neuron disease
*nfvPPA*: Nonfluent variant primary progressive aphasia
*ALS*: Amyotrophic lateral sclerosis
*123I­FP­CIT SPECT*: 123I-N-ω-fluoropropyl-2β-carbomethoxy-3β-(4-iodophenyl)nortropane SPECT
*SCAs*: Spinocerebellar ataxias
*FXTAS*: Fragile X tremor-ataxia syndrome
*MCP*: Middle cerebellar peduncle
*mtDNA*: Mitochondrial DNA
*POLG*: MtDNA polymerase gamma
*HSP*: Hereditary spastic paraplegias
*SPG7*: Spastic paraplegia type 7
*SPG11*: Spastic paraplegia type 11
*99mTc-TRODAT-1*: Technetium-99 m labeled tropane derivative
*SPRS*: Spastic Paraplegia Rating Scale
*MoCA*: Montreal Cognitive Assessment
